# Recent advances in PANoptosis research in kidney disease: mechanistic networks, pathological roles, and potential intervention strategies

**DOI:** 10.3389/fimmu.2026.1902148

**Published:** 2026-08-25

**Authors:** Shifan Yang, Xun Li, Chenming Zhang, Sicheng Ma, Yingda Zhou

**Affiliations:** 1 The Second Affiliated Hospital of Henan University of Traditional Chinese Medicine, Zhengzhou, China; 2 Department of Reproductive Andrology, Henan Provincial Hospital of Traditional Chinese Medicine, Zhengzhou, China

**Keywords:** acute kidney injury, diabetic nephropathy, kidney disease, PANoptosis, PANoptosome, precision, therapy

## Abstract

PANoptosis is an integrated inflammatory form of programmed cell death mediated by the PANoptosome, which coordinates the core molecular networks of pyroptosis, apoptosis, and necroptosis. In recent years, PANoptosis-related signaling has been implicated in a wide range of renal pathological processes, including sepsis-associated acute kidney injury, ischemia–reperfusion injury, drug- or toxin-induced nephropathy, diabetic nephropathy, chronic kidney disease, renal fibrosis, and clear cell renal cell carcinoma. Mechanistically, these processes may involve the coordinated activation of key molecules, including ZBP1, AIM2, RIG-I, RIPK1, CASP8, NLRP3, GSDMD/GSDME, and RIPK3/MLKL. However, most existing studies remain largely confined to marker co-expression, animal models, or bioinformatics-based analyses, while direct evidence of PANoptosome assembly and functional dependence remains limited. Notably, the predominant cell types, upstream stimuli, and biological consequences of PANoptosis vary substantially across disease contexts. In non-neoplastic kidney diseases, PANoptosis is commonly associated with inflammatory injury and fibrotic progression; in contrast, in clear cell renal cell carcinoma, PANoptosis-related molecular signatures are more frequently linked to prognostic stratification and prediction of immunotherapy response. Future studies should place greater emphasis on validating PANoptosome assembly, defining cell lineage-specific localization, establishing temporal causality, and confirming findings in clinical samples. Integrating single-cell multi-omics, spatial transcriptomics, and functional intervention models will help advance PANoptosis from a mechanistic concept toward precise kidney disease subtyping and network-based targeted therapeutic strategies.

This article aims to review recent advances in research on PANoptosis in kidney diseases. A literature search was conducted in April 2026 using the PubMed, Web of Science, and Embase databases. The search terms included “PANoptosis,” “kidney,” “renal,” “acute kidney injury,” “diabetic kidney disease,” “chronic kidney disease,” “renal fibrosis,” and “clear cell renal cell carcinoma.” A free-text search strategy was adopted, with terms restricted to the title and abstract fields to improve search specificity. Priority was given to basic research, translational studies, and review articles on PANoptosis and the cross-regulation of pyroptosis, apoptosis, and necroptosis. Articles unrelated to kidney diseases, duplicate publications, conference abstracts, and studies with insufficient supporting evidence were excluded. Studies reporting changes in markers of a single cell death pathway without validation of PANoptosome assembly or function were classified in this review as involving PANoptosis-related signaling rather than PANoptosis in the strict sense. The search period extended from database inception to April 2026.

## 1 Introduction

**Figure 1:**
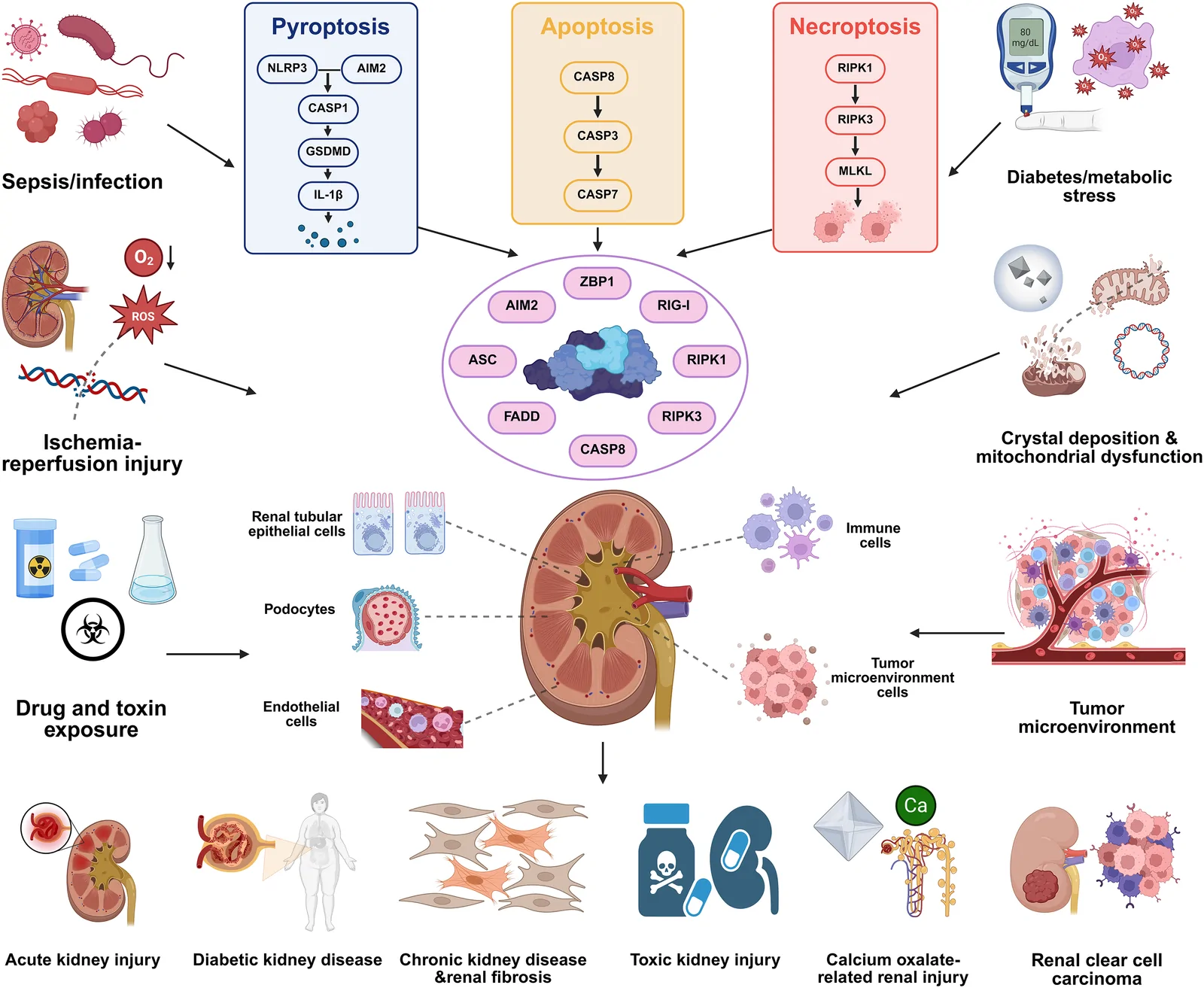
PANoptosis in kidney disease and potential intervention strategies.Schematic overview of PANoptosis, an integrated inflammatory cell death pathway that coordinates pyroptosis, apoptosis, and necroptosis through PANoptosome assembly. Key effector molecules and major renal cell types involved in this process are illustrated. Upstream triggers include infection, ischemia–reperfusion injury, drugs or toxins, hyperglycemia, crystal deposition, mitochondrial dysfunction, and the tumor microenvironment. Potential therapeutic interventions may target the PANoptosome hub, upstream immune sensors, mitochondrial homeostasis, or multi-target compounds.

Programmed cell death is a fundamental biological process that maintains tissue homeostasis and contributes to the initiation and progression of various diseases (1). Dysregulation of these pathways is implicated in a wide range of pathological conditions, particularly inflammatory disorders and organ injury. Traditionally, pyroptosis, apoptosis, and necroptosis were considered independent modes of cell death (2, 3). However, accumulating evidence indicates that, under complex pathological contexts, these pathways can engage in extensive crosstalk through shared molecular components and signaling platforms, thereby giving rise to PANoptosis—a coordinated and integrated form of inflammatory cell death (4, 5). The kidney is a metabolically active organ characterized by high energy demand, abundant mitochondrial content, and rich blood perfusion (6). It performs essential physiological functions, including filtration, reabsorption, ion transport, and local immune regulation. Owing to these features, the kidney is particularly susceptible to pathological insults such as ischemia–hypoxia, infection and inflammation, metabolic dysregulation, toxin exposure, and immune stress. Under such conditions, renal tubular epithelial cells, podocytes, endothelial cells, and infiltrating immune cells are prone to amplified inflammatory responses and concurrent activation of multiple programmed cell death pathways (7). Recent advances have further refined the concept of PANoptosis, defining it as an integrated cell death modality mediated by the assembly of the PANoptosome complex, which coordinates pyroptotic, apoptotic, and necroptotic signaling. In renal diseases, PANoptosis has emerged as a central mechanism linking innate immune activation, metabolic stress, and progressive tissue injury (8). Existing evidence has associated PANoptosis with a range of kidney disorders, including acute kidney injury, diabetic nephropathy, chronic kidney disease, and renal cell carcinoma (9, 10). This review systematically summarizes recent progress in understanding kidney diseases from the perspective of PANoptosis. It aims to deepen insights into the mechanisms underlying inflammatory renal injury and the interconnectedness of multiple cell death pathways, while also providing a mechanistic explanation for the limited efficacy of single-pathway therapeutic strategies. Ultimately, this perspective may facilitate a shift in kidney disease research and clinical intervention toward network-based and precision-targeted approaches (**Figure 1**).

## 2 The nature and molecular basis of PANoptosis

### 2.1 PANoptosis

PANoptosis is defined as an integrated form of inflammatory programmed cell death mediated by the PANoptosome(a multiprotein complex signaling platform). It exhibits molecular and morphological features of pyroptosis, apoptosis, and necroptosis, yet its biological nature and functional consequences cannot be fully explained by any single mode of cell death (11). The key feature of PANoptosis is not the mere coexistence or simultaneous activation of these three classical forms of programmed cell death. Rather, it lies in the reorganization of upstream receptors, adaptor and scaffold proteins, and effector molecules into a higher-order, unified platform for the execution of cell death under specific pathological stimuli and within an inflammatory microenvironment. Through shared receptors, scaffold molecules, and effector nodes, pyroptosis, apoptosis, and necroptosis are integrated into a lethal network characterized by multimodular interactions, functional redundancy, reciprocal compensation, and cascade amplification (12).

Evidence accumulated over the past several years has substantially expanded our understanding of the biological and pathological roles of PANoptosis. Early mechanistic investigations primarily focused on infectious and inflammatory diseases, particularly viral and bacterial infection models (13). In these contexts, pattern recognition receptors (PRRs), including ZBP1, AIM2, and NLRP3, were identified as upstream sensors that initiate PANoptosome assembly and recruit key effector molecules—such as caspase-8, RIPK1, and RIPK3—to form a multiprotein signaling complex (14). Among these receptors, ZBP1 is regarded as a central sensor driving PANoptosis, mediating inflammatory cell death through the RIPK1–RIPK3–caspase-8 signaling axis (12). Collectively, these findings established the conceptual basis for the PANoptosome as a higher-order molecular scaffold governing inflammatory cell death.

Subsequent studies have demonstrated that PANoptosis is not restricted to pathogen-induced inflammatory responses but is also widely involved in sterile pathological conditions, including ischemia–reperfusion injury, metabolic disorders, and tumor-associated immune remodeling (15). In these settings, diverse upstream stimuli—such as TNF-α/IFN-γ–driven cytokine storms, mitochondrial dysfunction, oxidative stress, and nucleic acid leakage—converge on shared signaling hubs, promoting caspase cascade activation, gasdermin cleavage (GSDMD/GSDME), and RIPK3–MLKL phosphorylation, thereby amplifying inflammatory cell death responses (16).

A key conceptual advance in recent literature is the evolution of PANoptosis from a model of parallel activation of multiple cell death pathways to a systems-level network reorganization process orchestrated by structured multiprotein complexes. As a central assembly platform, the PANoptosome coordinates the spatial and temporal integration of inflammatory sensing, signal transduction, and effector execution, thereby establishing a highly ordered cell death program under pathological conditions. This framework not only elucidates the mechanisms underlying the amplification of inflammation in complex diseases but also provides a theoretical basis for therapeutic resistance and persistent tissue injury. Nevertheless, further studies are required to clarify the *in vivo* assembly states of the PANoptosome across different tissues and disease contexts, as well as their functional relevance.

### 2.2 Composition and criteria for PANoptosis

**Figure 2:**
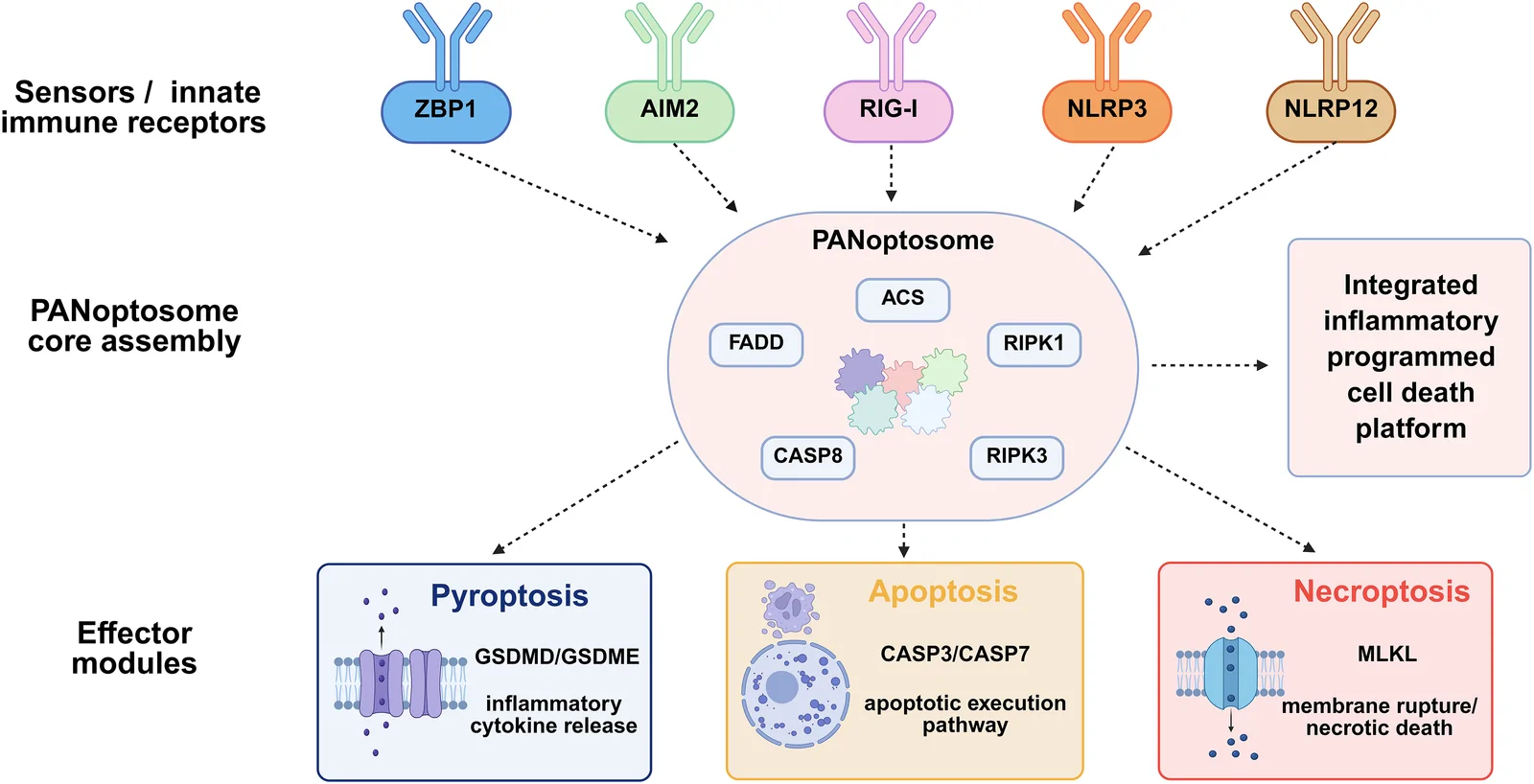
Schematic representation of the molecular network, effector modules, and diagnostic criteria of PANoptosis in kidney disease. Upstream inflammatory stimuli, damage-associated molecular patterns, oxidative stress, mitochondrial damage, and nucleic acid–sensing signals activate ZBP1, AIM2, RIG-I, NLRP3, and other pattern-recognition receptors. These signals promote PANoptosome assembly through adaptor and signaling molecules, including ASC, FADD, caspase-8, and RIPK1/RIPK3. The assembled PANoptosome integrates pyroptotic, apoptotic, and necroptotic pathways, thereby contributing to renal tubular injury, podocyte damage, endothelial dysfunction, inflammatory amplification, fibrosis progression, and modulation of the renal cancer immune microenvironment.

The PANoptosome is the central molecular scaffold platform that mediates PANoptosis. It is essentially a class of multiprotein complexes that integrate components from multiple cell death pathways (17), as shown in **Figure 2**. The most extensively studied PANoptosomes to date include the ZBP1-PANoptosome, AIM2-PANoptosome, RIPK1-PANoptosome, and NLRP12-PANoptosome (18). Although different PANoptosomes vary in receptor composition and inducing stimuli, they share the capacity to incorporate ASC, CASP8, FADD, RIPK1, RIPK3, caspase family members, and inflammasome-related components. Through this integration, inflammatory sensing, death signaling, and effector execution are coupled into a unified lethal platform (14, 15). Within this integrated network, CASP8 is widely recognized as a key molecular hub linking pyroptosis, apoptosis, and necroptosis. Meanwhile, RIPK1, which possesses both kinase activity and scaffold functions, plays a crucial role in driving necroptosis, promoting PANoptosome assembly, and integrating inflammatory signals (19). Consequently, effector modules such as NLRP3/GSDMD, CASP3/CASP7, and RIPK1/RIPK3/MLKL do not function in isolation. Instead, they constitute a highly nested and cross-activatable receptor–scaffold–effector network within a shared inflammatory context (20). Under conditions such as TNF-α/IFN-γ co-stimulation, cytoplasmic DNA or RNA accumulation, mitochondrial damage, oxidative stress, ion imbalance, and aberrant inflammasome activation, this network is accompanied by molecular events including GSDMD or GSDME cleavage, caspase-8/caspase-3 activation, and RIPK3/MLKL phosphorylation, reflecting the progression of terminal cell damage (21). It is important to note that the identification of PANoptosis should not rely solely on the simultaneous upregulation of markers such as GSDMD, cleaved caspase-3, RIPK3, or p-MLKL. More reliable evidence should include simultaneous activation of pyroptotic, apoptotic, and necroptotic effector molecules; evidence of interactions or complex assembly among key PANoptosome components, as demonstrated by co-immunoprecipitation, proximity ligation assays, or colocalization analysis; simultaneous attenuation of multiple cell death pathways following knockout of upstream receptors or scaffold molecules; and a stronger protective effect achieved by combined inhibition of multiple death pathways than by single-pathway inhibition. In the absence of evidence supporting complex assembly or genetic intervention, the term should be used cautiously as “PANoptosis-related signaling” or “PANoptosis-associated signaling” (2, 3, 22).

## 3 Mechanisms related to PANoptosis in acute kidney injury

Current research on PANoptosis in kidney diseases has primarily focused on acute kidney injury (AKI), with sepsis-associated AKI (S-AKI) and ischemia–reperfusion injury-induced AKI (IRI-AKI) representing the two contexts in which evidence is most concentrated (23). This focus is not only attributable to the prominent inflammatory responses, oxidative stress, and cell death that characterize AKI, but is also closely related to the central role of renal tubular epithelial cells as the core injury-executing unit. This feature facilitates the establishment of both *in vivo* and *in vitro* models and enables the validation of multi-pathway cell death mechanisms (24). Against this background, existing evidence suggests that PANoptosis may represent a shared inflammatory cell death program across different AKI subtypes.

### 3.1 Sepsis-associated acute kidney injury

Sepsis-associated acute kidney injury (S-AKI) is one of the renal disease contexts with relatively robust evidence supporting the involvement of PANoptosis (25). Existing studies indicate that PANoptosis is markedly activated in S-AKI, and the degree of activation is positively correlated with the severity of renal injury (26). Its occurrence is not driven by a single receptor or pathway but rather by multiple pathological factors, including cytokine storm, oxidative stress, mitochondrial dysfunction, and aberrant immune activation. Through the synergistic amplification of inflammatory and nucleic acid-sensing pathways, such as those mediated by NLRP3, AIM2, and ZBP1, PANoptosis may serve as a key pathological hub linking uncontrolled inflammation, metabolic disturbance, and cell death execution (27, 28). At the cellular level, proximal tubular epithelial cells are considered the primary executors of PANoptosis (29). In parallel, the NETs–dsRNA–TLR3 axis, the TNF–AIM2 signaling pathway, and local amplification loops involving immune and endothelial cells may further promote the transition from acute injury to persistent inflammation, tissue remodeling, and even fibrosis (30). Mechanistic studies have provided direct supporting evidence. For example, in S-AKI, EIF2AK2 promotes PANoptosis in renal tubular epithelial cells by upregulating AIM2 expression and activating downstream complex formation, thereby exacerbating renal dysfunction and tissue damage (31). Another study demonstrated that ZBP1 plays a significant amplifying role in S-AKI. Its increased expression is not only associated with intensified renal inflammation but also correlated with elevated levels of renal injury markers, including serum creatinine, blood urea nitrogen, KIM-1, and NGAL, suggesting that nucleic acid receptor-driven integrated inflammatory cell death may represent one of the key amplification mechanisms in infectious AKI (27, 32, 33). Taken together, PANoptosis likely occupies a central position in the continuous pathological cascade of “infection signal recognition–inflammatory transcriptional amplification–cell death execution” in S-AKI (27, 30, 33). Currently, evidence for PANoptosis in S-AKI mainly derives from LPS- or CLP-induced animal models, *in vitro* stimulation models of renal tubular epithelial cells, and limited human transcriptomic and histological data. Although these studies support the involvement of PANoptosis in the development of S-AKI, its temporal dynamics, cellular origins, and prognostic value in clinical patients remain to be further validated.

### 3.2 Ischemic-reperfusion acute kidney injury

Ischemia–reperfusion injury (IRI) is not only a major focus of acute kidney injury (AKI) research but also a key pathological basis for AKI onset and progression during surgical procedures, shock, kidney transplantation, and severe hypoperfusion (34). IRI is now recognized as a complex pathological event driven by the synergistic amplification of damage-associated molecular pattern release, innate immune activation, and multiple forms of cell death. Recent studies suggest that the STING–STAT1–ZBP1 axis may be involved in the initiation and amplification of PANoptosis-related signaling in IRI-AKI. This finding provides new mechanistic insight into the connection among DNA sensing, interferon signaling, and integrated cell death; however, its generalizability requires further validation in additional independent models and clinical samples (35–37). Furthermore, in transplantation and non-heart-beating donor models, the RIPK1 inhibitor necrostatin-1 has been shown to reduce PANoptosis after renal ischemia–reperfusion by targeting key nodes of the PANoptosome, thereby alleviating tissue damage and inflammatory responses (38). Notably, IRI can induce PANoptosis not only in renal tubular epithelial cells but also in endothelial cells through the ZBP1-PANoptosome, further expanding the cell lineage basis of ischemia–reperfusion-associated PANoptosis (36, 37, 39). Therefore, the introduction of the PANoptosis concept not only deepens our understanding of the pathological mechanisms underlying IRI but also provides a new theoretical framework and potential therapeutic directions for donor kidney protection and perioperative AKI intervention.

### 3.3 Drug- and toxin-induced acute kidney injury

Drug- and toxin-induced kidney injury has emerged as a rapidly expanding field in recent research on renal PANoptosis, providing important and insightful entry points for elucidating the underlying mechanisms (9). Regarding drug-induced kidney injury, studies have shown that amentoflavone can simultaneously inhibit ferroptosis and PANoptosis via an Nrf2-dependent mechanism, thereby alleviating cisplatin-induced kidney injury. This finding suggests that the cell death processes underlying cisplatin nephrotoxicity may not be dominated by a single death pathway, but rather form a more complex injury network through synergistic interactions, cross-regulation, and phenotypic conversion among different death pathways (9, 40). In aristolochic acid nephropathy, HDAC-mediated inhibition of PSTPIP2 has been shown to induce renal tubular PANoptosis (41). In toxic nephropathy, citrinin can induce renal PANoptosis by mediating mitochondrial dysfunction via the GSDMD-N/DRP1 axis, suggesting that mitochondrial abnormalities may be a key hub mechanism driving PANoptosis in toxic nephropathy (42). Furthermore, studies on TCE-induced renal injury indicate that PANoptosis occurs not only in renal tubular epithelial cells but also in renal vascular endothelial cells, and this process is regulated by the TNF-α/IFN-γ-IRF1 axis. Concurrently, neutralizing inflammatory factors or the combined inhibition of RIPK1 and caspase-8 can both alleviate renal injury, further supporting the synergistic integration of multiple cell death programs in toxic nephropathy (43). Therefore, PANoptosis in drug- and toxin-induced renal injury may exhibit strong stimulus-type dependence: the cisplatin model favors oxidative stress and Nrf2 regulation; the aristolochic acid model involves epigenetic regulation; whereas the citrinin and TCE models highlight mitochondrial damage and the synergistic action of inflammatory factors.

### 3.4 Acute kidney injury associated with trauma and systemic stress

Under conditions of severe stress, such as major trauma, the kidney is often among the first target organs to sustain acute injury, suggesting that PANoptosis may provide a compelling explanation for the pathogenesis of trauma-associated AKI (44). In early-stage burn-induced AKI, SUMOylation of ZEB1 has been shown to affect the expression and activation of multiple PANoptosis-related effector molecules, indicating that post-translational modifications play a crucial regulatory role in reprogramming cell death networks under severe stress conditions (45). Studies of crush syndrome-associated AKI further suggest that RIG-I, a key innate immune receptor involved in recognizing myoglobin-related DAMPs, mediates PANoptosis-related molecular alterations in renal tubular epithelial cells. Notably, inhibition of RIG-I significantly alleviates tubular injury, indicating that RIG-I may serve as a critical upstream node driving integrated inflammatory cell death in traumatic AKI (29). Mechanistically, RIG-I initiates this process by assembling a RIG-I PANoptosome composed of ASC, caspase-1, caspase-8, FADD, RIPK1, and RIPK3. Inhibition or knockdown of RIG-I not only disrupts the assembly of this complex but also attenuates multiple downstream cell death signals and enhances cell viability, thereby exerting a protective effect superior to that achieved by blocking a single cell death pathway (22). Collectively, these findings suggest that PANoptosis in trauma-associated AKI may depend more heavily on DAMP recognition and innate immune receptor activation. In particular, the assembly of RIG-I-associated complexes may represent a key mechanistic feature that distinguishes this subtype from infection- or ischemia-induced AKI models.

## 4 PANoptosis and chronic kidney disease and renal fibrosis

Diabetic nephropathy (chronic kidney disease, CKD) is one of the leading causes of chronic kidney disease and end-stage renal disease worldwide, characterized by persistent hyperglycemia, metabolic disorders, and abnormal changes in renal hemodynamics (46). Its pathogenesis involves multiple interrelated mechanisms, including oxidative stress, accumulation of advanced glycation end products (AGEs), chronic inflammation, mitochondrial dysfunction, and dysregulation of apoptotic pathways (47). Compared with AKI, research on PANoptosis in chronic kidney disease (CKD) has emerged relatively late, with early evidence mainly derived from transcriptomic, bioinformatic, and immune infiltration analyses (9, 48). More recent studies using models involving eCIRP/C23 and TMAO have begun to provide preliminary evidence for functional validation. Multiple studies have shown that PANoptosis-related genes are consistently dysregulated in CKD tissues and that these abnormalities are closely associated with chronic inflammation, immune cell infiltration, fibrosis, and disease progression (49). Mechanistic studies have demonstrated that eCIRP, a key DAMP molecule, sustains chronic inflammation and promotes renal fibrosis by inducing inflammatory responses and PANoptosis in renal tubular epithelial cells. Conversely, its antagonist peptide C23 mitigates RTEC injury, suppresses PANoptosis, and attenuates the fibrotic phenotype in the UUO model (50). Together with the persistent dysregulation of PANoptosis-related genes in CKD tissues, these findings suggest that PANoptosis may serve as a critical link connecting acute injury, chronic inflammatory maintenance, and fibrotic progression; however, its direct causal role remains to be further confirmed. In CKD models, TMAO-induced chronic kidney injury provides more direct evidence for the involvement of PANoptosis in disease progression (51). Long-term exposure to TMAO leads to renal functional decline and aggravated renal interstitial fibrosis, accompanied by the concurrent upregulation of molecules such as cleaved caspase-8, NLRP3, caspase-1, IL-1β, cleaved GSDMD, ZBP1, p-RIP3, and p-MLKL, whereas typical evidence of ferroptosis has not been observed. These findings suggest that, in the context of chronic injury mediated by metabolic uremic toxins, PANoptosis is more likely to continuously drive renal structural destruction and functional deterioration through a ZBP1-centered PANoptosome, rather than merely serving as a transient terminal event in inflammatory AKI (11, 51, 52).

In addition to animal models, multilayered genetic studies based on GWAS, mQTL, eQTL, SMR, and colocalization analyses have provided genetic epidemiological evidence for potential associations between PANoptosis-related genes and CKD risk (53). These studies suggest that genes such as PRKAR2A, HGF, CCND1, and MADD may be causally associated with CKD risk, indicating that PANoptosis is not merely a phenotypic event occurring at the terminal stage of tissue damage, but may also be embedded in the chronic pathological remodeling of CKD at the level of genetic regulation. This provides a theoretical basis for subsequent efforts to move beyond phenotypic associations toward the construction of causal pathways, precise disease subtyping, and targeted interventions (12, 53). Nevertheless, such statistical genetic evidence still requires functional validation in cellular and animal models, as well as in clinical samples. Furthermore, studies using models of persistent inflammation after burns have shown that healthy mice exhibit only subclinical renal alterations following severe burns, whereas adenine-induced CKD mice develop more pronounced renal dysfunction, exacerbated inflammatory responses, and enhanced cell death. These changes are accompanied by significant activation of caspase-1 and caspase-3 signaling, as well as ultrastructural abnormalities such as podocyte injury, cell membrane vesiculation, and membrane rupture (54). These observations suggest that, in the context of CKD, persistent systemic inflammation can markedly amplify PANoptosis-related renal injury. Conversely, dexamethasone treatment alleviates these injuries, further indicating that improvement of the inflammatory microenvironment may mitigate stress-induced deterioration of renal function by indirectly suppressing PANoptotic amplification (49, 55). Therefore, future CKD-related research should focus on determining whether PANoptosis acts as a driving force in the maintenance of chronic inflammation or represents a secondary molecular phenotype following tissue injury.

## 5 PANoptosis and diabetic nephropathy

Diabetic kidney disease (DKD) is a leading cause of chronic kidney disease and end-stage renal disease worldwide, characterized by persistent hyperglycemia, metabolic dysregulation, and abnormal renal hemodynamics (56). Its pathogenesis is driven by multiple interrelated mechanisms, including oxidative stress, accumulation of advanced glycation end products (AGEs), chronic low-grade inflammation, mitochondrial dysfunction, and dysregulation of programmed cell death pathways (57). DKD is also among the most extensively studied and rapidly advancing subtypes of chronic kidney disease within the field of PANoptosis research (58). Existing studies have primarily focused on two cellular compartments: podocytes and proximal tubular cells (59). Accordingly, PANoptosis offers a new integrative framework for understanding the mutual amplification of glomerular and tubulointerstitial injury in DKD. Podocyte injury and loss constitute a critical pathological basis for DKD progression.

### 5.1 Receptor signaling in podocyte injury and death

Existing studies indicate that TRAIL and its receptor DR5 are significantly upregulated in podocytes in DKD and are correlated with disease severity. Further evidence from single-cell sequencing, as well as *in vitro* and *in vivo* functional experiments, demonstrates that the TRAIL/DR5 axis can drive PANoptosis-related signaling in podocytes, whereas DR5-Fc intervention alleviates proteinuria and glomerular injury (58, 60). These findings suggest that podocyte death in a hyperglycemic environment is not merely characterized by enhanced classical apoptosis, but is more likely to represent an integrated inflammatory cell death process mediated by death receptors.

### 5.2 Damage to renal tubular epithelial cells and transcriptome regulation

In addition to glomerular injury, tubular injury is also a central component of DKD initiation and progression (59). Multiple integrated transcriptomic analyses indicate that PANoptosis-related molecules are consistently dysregulated in DKD tubules. Further studies combining WGCNA, machine learning, Nephroseq, and single-cell sequencing have identified 31 PANoptosis-related differentially expressed genes that are dysregulated in DKD. Among them, TAP1, CASP1, and PRKX were identified as key hub genes; these molecules are not only associated with reduced GFR but are also predominantly expressed in proximal tubular cells (61, 62). Consistently, these molecules are persistently upregulated in high-glucose-treated tubular cells, DKD mouse kidney tissues, and human DKD biopsy samples. Their functions are mainly enriched in processes such as necroptosis, NOD-like receptor signaling, inflammasome activation, cytokine production, extracellular matrix remodeling, and chemokine signaling (5, 58). This evidence suggests that PANoptosis in DKD is not an isolated cell death phenomenon, but is deeply embedded in the network of renal tubular inflammation, immune infiltration, and fibrotic remodeling. It may therefore serve as a critical pathological module and potential therapeutic target driving disease progression. Therefore, PANoptosis in DKD may occur in at least two cellular contexts. The first is death receptor-mediated PANoptosis in podocytes, represented by the TRAIL/DR5 axis. The second is a PANoptosis-related transcriptional regulatory program in proximal tubular cells, associated with hyperglycemic stimulation, inflammasome activation, NOD-like receptor signaling, and immune infiltration. These two mechanisms may contribute, respectively, to the development of proteinuria and to the tubulointerstitial inflammatory-fibrotic process.

### 5.3 Regulation mediated by non-coding RNA

From a broader regulatory perspective, non-coding RNAs may also participate in the regulation of DKD-associated PANoptosis (63). Current review evidence suggests that non-coding RNAs may influence the cross-activation of pyroptosis, apoptosis, and necroptosis in DKD by regulating molecules such as the Bcl-2 family, caspases, IL-1β/IL-18, and RIPK1/RIPK3/MLKL (64, 65). However, functional evidence demonstrating that non-coding RNAs directly regulate PANoptosome assembly or induce PANoptosis remains lacking. Therefore, non-coding RNAs are better regarded as regulatory clues for PANoptosis-related microenvironmental changes and disease progression, rather than as independent mechanistic evidence. In future studies, non-coding RNAs may also serve as early diagnostic biomarkers and targets for precision interventions in DKD-associated PANoptosis.

## 6 PANoptosis and renal injury of specific etiology

In addition to AKI and DKD, the role of PANoptosis in other specific renal pathological conditions is increasingly gaining attention (69). Existing studies indicate that in calcium oxalate (CaOx) crystal-associated kidney injury, crystal-induced epithelial damage, inflammatory responses, and cell death constitute critical pathological foundations for stone formation and recurrence. Salidroside has been shown to alleviate CaOx-related kidney injury and inflammatory responses by inhibiting PANoptosis, suggesting that crystal-induced renal parenchymal damage can be further amplified through an integrated inflammatory cell death program (70). Concurrently, mounting evidence suggests that mitochondrial dysfunction may represent a critical common basis for PANoptosis induction. In mitochondrial-related studies, mPTP opening, mtDNA release, and DRP1-mediated mitochondrial dynamic imbalance are considered key upstream events initiating PANoptosis (66, 67). These toxicological studies underscore the significance of mitochondrial abnormalities in promoting integrated cell death. Furthermore, although evidence that disulfiram regulates PANoptosis via modulation of mitochondrial permeability does not originate from renal disease models, the kidney’s high dependence on mitochondrial homeostasis suggests that this mechanism may provide valuable insights for exploring mitochondrial-protective strategies in renal injury (68, 71, 72). Nevertheless, its efficacy and safety in renal disease require further validation using dedicated experimental models. Overall, these specific pathogenic models suggest that, in addition to infection and ischemia, crystal deposition, toxic metabolites, and disruption of mitochondrial homeostasis may act as non-classical stimuli that trigger PANoptosis-related renal damage.

## 7 PANoptosis and renal clear cell carcinoma

The biological significance of PANoptosis in the kidney extends beyond non-neoplastic injury (73). In clear cell renal cell carcinoma (ccRCC), multiple bioinformatics studies have demonstrated that PANoptosis-associated gene and miRNA signatures can be used not only for patient stratification, prognostic assessment, and immune infiltration analysis, but also for identifying tumor heterogeneity and predicting immune-related outcomes (74, 75). This context-dependent role indicates that PANoptosis cannot be simplistically categorized as either “activated” or “inhibited.” In non-neoplastic kidney diseases, PANoptosis primarily represents a pathological process that requires inhibition, whereas in renal cancer, it may serve a dual function as both a prognostic marker and a potential therapeutic target (67, 76). It should be noted that most ccRCC studies have constructed prognostic models or evaluated immune infiltration patterns based on PANoptosis-related genes. These analyses reflect PANoptosis-associated molecular signatures but do not necessarily indicate the presence of classical PANoptosome assembly or execution of PANoptosis within tumor cells (77, 78). Therefore, future studies should integrate multiplex immunofluorescence, spatial transcriptomics, and functional experiments in tumor tissues to verify the cellular origin and biological effects of these molecular signatures.

## 8 Conclusion

The existing literature remains largely descriptive and disease-specific, and has not yet been integrated into a unified mechanistic framework. From a systems biology perspective, PANoptosis in kidney disease can be conceptualized as a hierarchical, stimulus-driven network of inflammatory cell death, in which heterogeneous upstream injury signals converge on conserved intracellular sensing modules, scaffold signaling platforms, and downstream effector pathways.

At the input and convergence levels, diverse renal stressors—including pathogen-associated molecular patterns (PAMPs) in sepsis, damage-associated molecular patterns (DAMPs) associated with ischemia–reperfusion injury and trauma, metabolic stress in diabetic nephropathy, exposure to crystals and toxins, and signals from the tumor microenvironment—are primarily sensed by a constrained network of pattern recognition receptors. Key components of this network include ZBP1, AIM2, RIG-I, NLRP3, and the cGAS–STING pathway. These upstream signals are subsequently integrated and amplified by adaptor and scaffold proteins, most notably RIPK1, CASP8, ASC, and FADD, which collectively constitute the central hub for PANoptosome assembly and signal integration.

At the execution level, PANoptosis is characterized by the coordinated activation of three mechanistically interconnected and partially redundant cell death programs: pyroptosis (GSDMD/GSDME–inflammasome axis), apoptosis (CASP8–CASP3 cascade), and necroptosis (RIPK1/RIPK3/MLKL pathway). Importantly, these effector modules are not activated independently; instead, they exhibit dynamic coupling and reciprocal regulation within a unified inflammatory signaling platform. When one pathway is inhibited, compensatory activation of the others can sustain cell death progression through signal reinforcement and amplification.

Despite this shared architectural framework, PANoptosis in different kidney diseases exhibits marked context-dependent heterogeneity. In acute kidney injury, it is primarily driven by acute inflammatory bursts and rapid DAMP release, with renal tubular epithelial cells serving as the principal effector population. In contrast, diabetic nephropathy and chronic kidney disease are characterized by sustained metabolic stress and chronic low-grade inflammation, leading to persistent activation of PANoptosis-associated transcriptional programs and progressive fibrotic remodeling. Drug- and toxin-induced kidney injury exhibits stimulus-specific mechanisms; for example, cisplatin-induced nephrotoxicity is driven by oxidative stress, aristolochic acid injury is closely associated with epigenetic dysregulation, and mitochondrial dysfunction underlies additional toxic injury models. In trauma and systemic stress conditions, innate immune receptors such as RIG-I play dominant roles in early signal initiation, whereas in renal cell carcinoma, PANoptosis-related signatures reflect immune microenvironment remodeling and tumor immune stratification rather than classical execution of cell death.

## 9 PANoptosis targeted intervention: from single-pathway inhibition to network-level regulation

The translational significance of PANoptosis lies in its ability to mechanistically explain why isolated inhibition of caspases, inflammasomes, or RIPK1 often yields only limited protective effects in various kidney diseases. The fundamental reason is that pyroptosis, apoptosis, and necroptosis are integrated at the PANoptosome level; as long as the upstream lethal network remains activated through bypass pathways, cells may continue to undergo terminal injury via alternative branches of cell death. Therefore, intervention strategies targeting PANoptosis should emphasize network-level regulation rather than being restricted to the inhibition of a single pathway or target (19, 20, 69, 79). The key characteristics of PANoptosis-related signaling pathways and potential therapeutic strategies in different kidney diseases are summarized in **Table 1**. Based on current evidence, PANoptosis-related intervention strategies can be broadly classified into four categories. The first category comprises central complex inhibition strategies, exemplified by Necrostatin-1, which primarily reduces integrated cell death by inhibiting key PANoptosome nodes such as RIPK1. The second category targets upstream sensors or inflammatory sources, such as AIM2, RIG-I, eCIRP, and NETosis, thereby blocking PANoptosis initiation at the level of danger signal input. The third category involves mitochondrial-protective strategies, represented by disulfiram, activators of antioxidant pathways, and regulatory approaches targeting mPTP, DRP1, and mtDNA. These strategies maintain mitochondrial homeostasis while simultaneously interfering with multiple cell death pathways. The fourth category consists of natural product-based or multi-target regulatory strategies, represented by compounds such as salidroside and amentoflavone, which combine anti-inflammatory, antioxidant, and multi-pathway synergistic regulatory effects. Overall, although PANoptosis-targeted therapy remains in the preclinical exploratory stage, it holds promise for directly blocking the co-assembly and cascade amplification of multiple cell death pathways. Therefore, this strategy is theoretically better aligned with the pathological nature of complex inflammatory injury in kidney disease and has considerable translational potential (36, 69, 80). At present, evidence supporting these interventions mainly derives from cellular and animal studies, whereas clinical trials validating their efficacy in renal diseases remain lacking. Future research should therefore systematically evaluate the target specificity, renal safety, and therapeutic window of candidate drugs, as well as their potential effects on host immune defense.

**Table 1:** Key characteristics of PANoptosis-related signaling pathways and potential intervention strategies in various kidney diseases.

Disease/model type	Main cell types	Key molecules	Major triggers	Potential interventions	Supporting evidence
Sepsis-associated acute kidney injury (S-AKI) (21–23)	Proximal tubular cells; endothelial/immune cells	AIM2, ZBP1, NLRP3, CASP1/3/8, GSDMD, RIPK3/MLKL	PAMPs/DAMPs, NETs, cytokine storm, ROS	Targeting AIM2/ZBP1/NETs; combined inhibition of multiple death pathways	LPS/CLP animal models; tubular epithelial cell experiments; correlation; limited human transcriptomics
Ischemia-reperfusion injury-associated AKI (IRI-AKI) (28–30)	Tubular epithelial cells; renal vascular endothelial cells	STING–STAT1–ZBP1, RIPK1/3, MLKL, CASP8, GSDMD	Ischemia/hypoxia, reperfusion-induced ROS, DAMPs, cytosolic DNA	RIPK1 inhibition; targeting the STING/ZBP1 axis; donor kidney protection	Rat IRI models; transplant and non-heart-beating donor models; ZBP1-PANoptosome assembly evidence
Cisplatin-induced drug-related AKI (34, 35)	Tubular epithelial cells	Nrf2, CASP3/8, GSDMD, RIPK3/MLKL, ferroptosis-related molecules	DNA damage, oxidative stress, mitochondrial injury	Nrf2 activation; antioxidant therapy; multi-pathway cell death modulation	Mouse nephrotoxicity models; ferroptosis–PANoptosis interaction; Nrf2-dependent protection
Aristolochic acid-associated kidney injury (36)	Tubular epithelial cells	HDAC, PSTPIP2, CASP1/3/8, GSDMD, RIPK3/MLKL	Toxic exposure, epigenetic dysregulation, chronic inflammation	HDAC inhibition; restoration of PSTPIP2-mediated protection	Animal nephrotoxicity models; HDAC-mediated epigenetic regulation; functional rescue experiments
Citrinin-induced toxic kidney injury (37)	Tubular epithelial cells	GSDMD-N, DRP1, CASP1/3/8, RIPK3/MLKL	Mycotoxin exposure, mitochondrial dynamics imbalance, ROS	DRP1 inhibition; mitochondrial protection; antioxidant intervention	Oxidative stress assays; mitochondrial dynamics dysfunction; pharmacologic inhibition studies
Trichloroethylene-associated kidney injury (38)	Tubular epithelial cells; vascular endothelial cells	TNF-α, IFN-γ, IRF1, RIPK1, CASP8, GSDMD, RIPK3/MLKL	Chemical toxicant exposure, TNF-α/IFN-γ synergy, inflammatory amplification	Cytokine neutralization; combined RIPK1/CASP8 inhibition	Rodent exposure models; dual-cell (tubular/endothelial) validation; inflammatory axis activation
Burn-associated AKI (40)	Tubular epithelial cells; podocytes; immune cells	ZEB1, SUMOylation-related molecules, CASP1/3, GSDMD, RIPK3/MLKL	Systemic inflammation, circulating inflammatory mediators, oxidative stress	Modulation of ZEB1 SUMOylation; anti-inflammatory and antioxidant strategies	Burn AKI models; SUMOylation regulation of cell death; caspase activation assays
Crush syndrome-associated AKI (41)	Tubular epithelial cells	RIG-I, ASC, FADD, CASP1/8, RIPK1/3, GSDMD, MLKL	Myoglobin release, DAMP accumulation, RIG-I-mediated innate immune sensing	RIG-I inhibition; blockade of PANoptosome assembly	Rhabdomyolysis models; RIG-I PANoptosome assembly; knockdown improves renal function markers
Chronic kidney disease and renal fibrosis (43–45)	Tubular epithelial cells; podocytes; immune/stromal cells	eCIRP, ZBP1, CASP8, NLRP3, GSDMD, p-RIPK3/p-MLKL	Chronic inflammation, persistent DAMP exposure, immune infiltration, profibrotic microenvironment	C23 peptide; ZBP1 pathway inhibition; combined anti-inflammatory and antifibrotic therapy	UUO fibrosis model; transcriptomics; GWAS/eQTL integration; eCIRP functional blockade
TMAO-induced chronic kidney injury (36)	Tubular epithelial cells; stromal cells	ZBP1, CASP8, NLRP3, CASP1, GSDMD, RIPK3/MLKL, IL-1β	TMAO accumulation, uremic toxin burden, chronic low-grade inflammation	Reduction of TMAO burden; targeting ZBP1; anti-inflammatory therapy	Metabolic CKD models; multi-omics pathway activation; fibrosis correlation
Diabetic kidney disease: podocyte injury (51)	Podocytes	TRAIL/DR5, CASP8/3, GSDMD, RIPK3/MLKL	Hyperglycemia, oxidative stress, death receptor signaling	DR5-Fc intervention; blockade of the TRAIL/DR5 axis; glomerular filtration barrier protection	Single-cell RNA-seq; human biopsy samples; mouse models; WGCNA + ML analysis
Diabetic kidney disease: proximal tubular injury (52, 53)	Proximal tubular epithelial cells	TAP1, CASP1, PRKX, NLR-related molecules, chemokines	Hyperglycemia, NOD-like receptor signaling, inflammasome activation, immune infiltration	Targeting the TAP1/CASP1/PRKX network; inflammasome inhibition	Crystal-induced AKI models; inflammasome activation; oxidative stress assays
Diabetic kidney disease: non-coding RNA-mediated regulation (55, 56)	Podocytes; tubular epithelial cells; immune microenvironment	miR-21, miR-141, miR-30e, Bcl-2 family, caspases, RIPK1/3, MLKL	Hyperglycemia-induced ncRNA dysregulation, ROS, inflammatory remodeling	Candidate biomarkers/regulatory entry points; further causal validation required	Mitochondrial permeability assays; DRP1 inhibition studies; cross-model consistency
Calcium oxalate crystal-associated kidney injury (58)	Tubular epithelial cells	GSDMD, caspase-related molecules, RIPK3/MLKL, inflammatory cytokines	Crystal deposition, epithelial injury, ROS, crystal-induced inflammation	Salidroside; anti-inflammatory and antioxidant therapy; cell death network modulation	Crystal-induced AKI models; inflammasome activation; oxidative stress assays
Mitochondrial dysfunction-associated kidney injury (59, 60)	Tubular epithelial cells	mPTP, mtDNA, DRP1, GSDMD-N, CASP8, RIPK3/MLKL	mPTP opening, mtDNA leakage, mitochondrial fission, ROS accumulation	Mitochondrial protection; DRP1 modulation; mPTP blockade	Mitochondrial permeability assays; DRP1 inhibition studies; cross-model consistency
Clear cell renal cell carcinoma (ccRCC) (66–68)	Tumor cells; immune/microenvironmental cells	PANoptosis-related gene/miRNA signatures, immune checkpoints, inflammatory infiltration markers	Tumor inflammatory microenvironment, immune infiltration, metabolic reprogramming	Prognostic and immunotherapy stratification; exploration of combination therapies	TCGA/GEO bioinformatics; prognostic modeling; immune infiltration analysis

## 10 Current challenges and future prospects

Although substantial progress has been made in the study of PANoptosis in kidney disease, several key questions remain unresolved. First, evidence regarding direct PANoptosome assembly, critical dependency nodes, and their temporal causal relationships remains insufficient. Therefore, future studies should integrate approaches such as genetic knockout, co-immunoprecipitation, temporal lineage tracing, single-cell multi-omics, and spatial transcriptomics to better distinguish concurrent activation of multiple forms of cell death from bona fide PANoptosis, as well as to elucidate its cell lineage specificity and stage-dependent differences across diverse disease contexts. Furthermore, given that the core cell types driving PANoptosis, together with its biological effects and clinical significance, differ among AKI, DKD, and renal cancer, future research and translational applications should place greater emphasis on refined stratification according to disease context, disease stage, and target cell population. Meanwhile, histological diagnostic criteria, circulating biomarker systems, and drug safety evaluation frameworks should be further refined to facilitate the clinical translation of PANoptosis toward precision medicine.
